# HPV-associated oropharyngeal cancer: in search of surrogate biomarkers for early lesions

**DOI:** 10.1038/s41388-023-02927-9

**Published:** 2024-01-08

**Authors:** Yvonne X. Lim, Nisha J. D’Silva

**Affiliations:** 1 Department of Periodontics and Oral Medicine, University of Michigan School of Dentistry, 1011 N. University Ave, Ann Arbor, MI USA; 2 Department of Pathology, University of Michigan Medical School, Ann Arbor, MI USA; 3 Rogel Cancer Center, University of Michigan, 1500 E Medical Center Dr, Ann Arbor, MI USA

**Keywords:** Head and neck cancer, Biomarkers

## Abstract

The incidence of oropharyngeal cancer (OPSCC) has escalated in the past few decades; this has largely been triggered by high-risk human papillomavirus (HPV). Early cancer screening is needed for timely clinical intervention and may reduce mortality and morbidity, but the lack of knowledge about premalignant lesions for OPSCC poses a significant challenge to early detection. Biomarkers that identify individuals at high risk for OPSCC may act as surrogate markers for precancer but these are limited as only a few studies decipher the multistep progression from HPV infection to OPSCC development. Here, we summarize the current literature describing the multistep progression from oral HPV infection, persistence, and tumor development in the oropharynx. We also examine key challenges that hinder the identification of premalignant lesions in the oropharynx and discuss potential biomarkers for oropharyngeal precancer. Finally, we evaluate novel strategies to improve investigations of the biological process that drives oral HPV persistence and OPSCC, highlighting new developments in the establishment of a genetic progression model for HPV + OPSCC and in vivo models that mimic HPV + OPSCC pathogenesis.

## Introduction

**Fig. 1:**
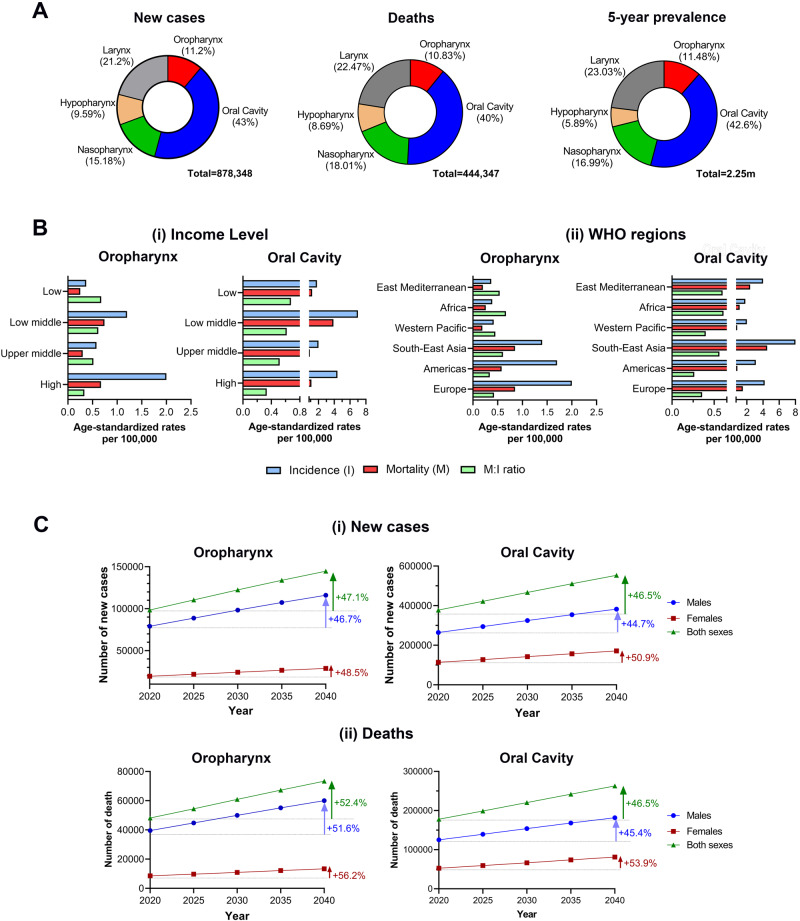
Comparison of oropharyngeal and oral cavity cancers. **A** Percentage by subsites of estimated annual new cases, deaths and 5-year prevalence of HNSCC in 2020. HNSCC is a group of tumors in the oral cavity and pharynx (oral cavity, oropharynx, nasopharynx, hypopharynx) and larynx. **B** Age-standardized incidence and mortality rates per 100,000 according to: (i) income classification as determined by World Bank Group; and (ii) World Health Organization (WHO) regions. **C** Estimated global number and percentage increase of: (i) annual newly diagnosed cases, and (ii) deaths for patients with oropharyngeal and oral cavity cancer from 2020 to 2040. All data related to incidence, mortality, and prevalence were obtained from Global Cancer Observatory 2020 [101–105]. Figures were plotted using GraphPad Prism 9. Data accessed on 23 May, 2023.

**Fig. 2:**
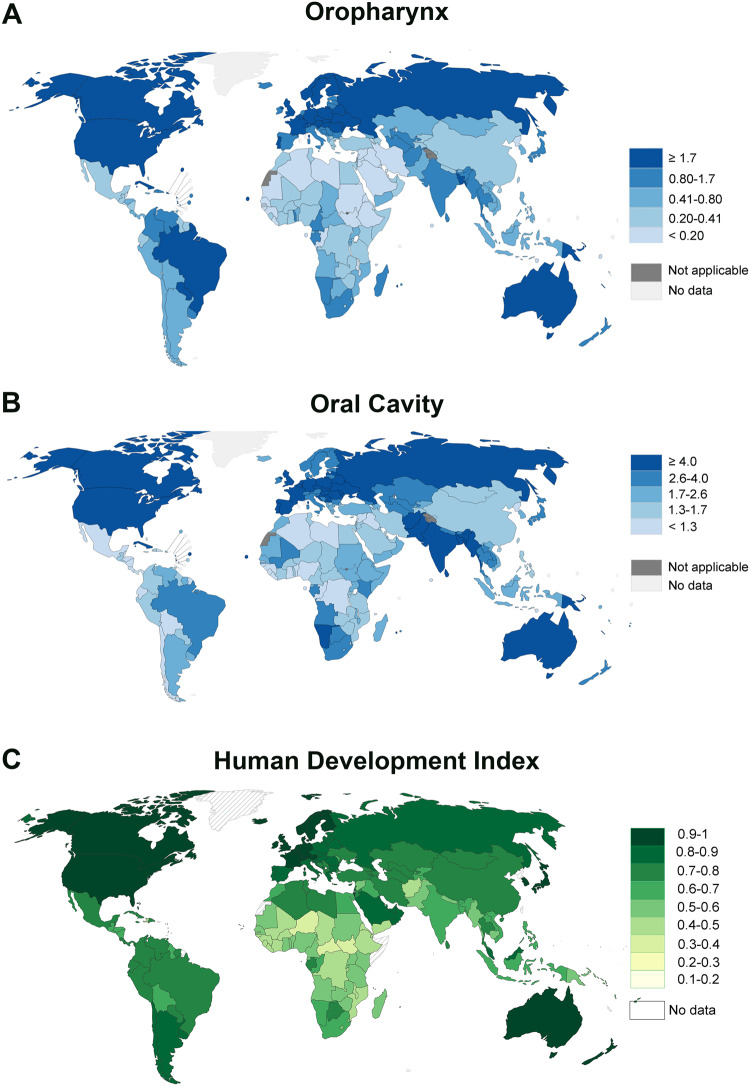
Incidence of oropharyngeal and oral cavity cancers in comparison to the human development index. World map depicting age-standardized incidence rates per 100,000 for oropharyngeal (**A**) and oral cavity (**B**) cancer. **C** World map showing Human Development Index classification according to United Nations 2019 Human Development Report. **C** was adapted by Our World in Data with permission^5^. All data related to incidence and mortality were obtained from Global Cancer Observatory 2020 [101–105]. Data accessed on 23 May, 2023.

Oropharyngeal cancers occur in the tonsils, base of tongue, soft palate, and posterior pharyngeal wall. Like other head and neck squamous cell carcinomas (HNSCC), over 95% of oropharyngeal cancers arise from squamous cells in the mucosal lining epithelium and are known as oropharyngeal squamous cell carcinoma (OPSCC) [1, 2]. Globally, OPSCC accounts for 11% of all HNSCCs; this incidence falls significantly behind oral cavity cancer, the predominant subsite for HNSCCs (over 40%) (Fig. 1A). Nonetheless, the clinical and scientific focus on OPSCC is due to its rapidly increasing prevalence especially in high-income and developed countries [3–6] (Figs. 1B, C and 2). Since the global incidence and mortality rates of OPSCC are expected to rise by approximately 50% in the next 20 years (Fig. 1C), there is a dire need to better understand the disease course to aid in prevention, early detection, and treatment.

Historically, OPSCC was associated with tobacco and alcohol consumption [3]. However, overwhelming evidence now points toward high-risk human papillomaviruses (HPV) as the main culprits driving the dramatic rise in incidence of OPSCC [3, 4, 7]. In the United States, the proportion of OPSCCs that are positive for HPV (HPV + OPSCC) increased from 16% in the 1980s to more than 70% in the early 2000s [3, 8]. In 2011, it was predicted that OPSCC would surpass cervical cancer to become the most common HPV+ malignancy in the United States by 2020 [3]; this occurred in 2012 [9]. In the United Kingdom, HPV + OPSCC surpassed cervical cancer in 2016 [9]. Similar trends were observed in European countries including Germany, Sweden, and Denmark [6, 10, 11]. In contrast, there is a decline in HPV-negative [HPV(-)] OPSCC [3, 4]. Among all head and neck regions, HPV-induced tumorigenesis favors the oropharynx; the fraction of HPV+ cancers remains markedly low at non-oropharyngeal subsites [12–14]. Moreover, HPV + OPSCC is more common among white males and non-smokers [14], but there is a rising trend in women and non-white people as well [15, 16]. Several studies revealed that patients with HPV + OPSCC who have tobacco exposure are at higher risk for recurrence [17, 18]. Patients with HPV + OPSCC are generally younger than those with HPV(-) OPSCC [14], although an increase in elderly patients has been reported [19–21]. A recent study on a cohort of over 3000 healthy adults in the United States reported that oral HPV DNA is most prevalent in older males aged 51–60 years [22]. An earlier study also reported that males aged 60–64 years had higher oral HPV prevalence compared to younger age groups [23]. The reason for the increased incidence with age is unclear, but could be due reactivation of latent infection, age-related immune compromise, and differences in sexual behaviors across age cohorts [23]. The favorable survival associated with HPV positivity persists in older patients with OPSCC, although the prognosis is worse than younger patients [24, 25].

While positivity for HPV DNA or its surrogate marker p16 protein confers superior prognosis to OPSCC [17, 26], recent attempts to reduce treatment intensity for patients with HPV + OPSCC were unsuccessful [27, 28]. Standard treatment leads to substantial morbidities [29–31]. Notably, a significant proportion of HPV+ OPSCCs progress or persist even after receiving standard aggressive therapy [17, 26, 32]. Although detection of precancerous lesions generally facilitates timely intervention and prevention of cancer, for OPSCC this is precluded by the lack of clearly defined precursor lesions [33–35]. The natural history of oral HPV infection and its multistep progression to cancer also remains poorly understood. HPV + OPSCC is usually undetected at early stages and frequently diagnosed after enlargement and spread to regional lymph nodes [36, 37]. Novel diagnostic strategies are urgently needed to overcome these limitations.

Here, we summarize the current literature on oral HPV infection and persistence that are believed to be the precursors of HPV + OPSCC. We also evaluate the shortcomings of current diagnostic tools to detect premalignant and early-stage malignant lesions of HPV + OPSCC and discuss the ongoing attempts to overcome these challenges. We envision that this review will spur efforts to decipher the molecular basis underlying multistep development and progression for HPV + OPSCC and will facilitate the design of diagnostic and therapeutic strategies for premalignant and malignant HPV+ lesions in the oropharynx.

## Acquisition and persistence of oral HPV

### Transmission of oral HPV

HPV is a human pathogen that infects epithelium in the cervix, oropharynx, oral cavity, and anogenital tract. Multiple HPV genotypes have been identified in the oral and oropharyngeal mucosa, of which, HPV 16 is the most prevalent; [22, 23, 38] this could explain why HPV16 is the leading genotype causing OPSCC [39–41]. The natural history of oral HPV infection is poorly understood compared to cervicogenital HPV [42–44]. This could be because oral HPV is detected at lower frequencies [23, 42, 43] and tends to be more transient [44] than cervicogenital HPV infection. Although oral HPV infection is more common in men than women [23], concomitant cervicogenital infection in women or anogenital infection in men increases the chance of oral HPV in both men and women alike [45, 46]. The specific mode of oral HPV transmission is unclear, but may be through oral-oral [46, 47], oral-genital [46, 48], and oral-anal contacts [49]. Therefore, infection of oral HPV is strongly correlated with the number of lifetime sexual partners engaging in deep kissing, orogenital, and vaginal sex [46, 47, 50, 51]. In a recent study, oral sex debut at a younger age also increases the odds of developing HPV + OPSCC, even after accounting for the number of oral and vaginal sex partners [51]. Non-sexual modes of transmission of oral HPV are also possible; perinatal transmission from mothers to their babies may occur either during pregnancy via the umbilical cord or placenta, during birth through the infected birth canal, or after birth through breast milk [52]. It is unclear if modes of transmission differ between HPV genotypes. In infants, the amount of DNA from high-risk genotypes of oral HPV peaks at 6 months and gradually declines at 24 months [53]. Children with persistent HPV infections are highly likely to suffer from chronic HPV-related diseases such as tonsillar hyperplasia and tonsillitis, and thus represent a source of silent and persistent oral HPV [54].

### Overview of HPV life cycle

The life cycle of HPV initiates when virus infects basal epithelial cells by binding heparan sulfate proteoglycans and then internalizes via actin-mediated endocytosis [55, 56]. Viral L2 and host cytoplasmic proteins, including retromer and γ-secretase, facilitate viral particle movement to the trans-Golgi network (TGN) [57–62]. L2 viral capsid protein associates with viral DNA (vDNA) to form a L2/vDNA complex that is encapsulated in a membrane-bound vesicle within the TGN [63]. In contrast, the majority of the L1 capsid protein dissociates from the L2/vDNA complex and is lysosomally degraded [64]. Following nuclear envelope breakdown during mitosis, the L2/vDNA complex enters the nucleus where it associates with chromosomes and remains in vesicles throughout mitosis [65–67]. Transit of the L2/vDNA complex from the Golgi to mitotic chromosomes is mediated by cyclin-dependent kinase 1- and polo like kinase 1-induced phosphorylation of L2 during G2/M transition [68]. Interaction with these two key kinases likely triggers a conformational change of L2 to induce tethering to chromatin [68]. The nuclear envelope re-forms upon completion of mitosis, the HPV genome is released from its transport vesicle [67] and undergoes three distinct stages of DNA replication: genome amplification, episomal maintenance, and vegetative amplification. Viral E1 and E2, expressed early in the HPV life cycle, encode viral helicase and ori-recognition proteins, respectively. Together, they initiate HPV genome amplification by recruiting and assembling host DNA replication machinery [69, 70]. Once the infected cell has attained a low basal viral copy number (~50–100 copies/cell), it proceeds to the maintenance phase where the number of viral episomes is kept constant until host cell differentiation [69].

When an infected keratinocyte divides, the HPV genome replicates and divides equally between the two daughter cells. This process is facilitated by the E2 protein that tethers to mitotic chromosomes to ensure equal distribution of the viral genome [71, 72]. One daughter cell becomes the basal epithelial cell while the other, moves into the upper epithelial layers To differentiate. Vegetative DNA amplification occurs in infected and differentiated cells in suprabasal epithelial layers, where viral genome copy number may increase to ~10,000 copies/cell [73]. The switch from episomal maintenance to vegetative DNA amplification is not fully understood but appears to be dependent on keratinocyte differentiation [74]. HPV *E6/E7* transcripts are expressed at low levels in proliferating basal keratinocytes but are transcriptionally activated in terminally differentiated cells [75, 76]. A key function of E6 is inactivation of the p53 pathway via binding with E6AP ubiquitin ligase [77]. Other cellular targets of high-risk E6 include MYC, FADD, TNFR1, TERT, and PDZ-containing proteins [78, 79]. In contrast, E7 inhibits Rb in turn driving E2F activity to promote transcription of cell cycle regulatory genes such as *p16*^*INK4A*^, cyclin A, and cyclin D [80, 81]. An alternative mechanism for E7 to induce *p16*^*INK4A*^ expression is through global demethylation of histone H3K27 [82]. This leads to re-entry of suprabasal differentiated keratinocytes into the cell cycle, a significant step towards unrestrained cell proliferation. E7 also suppresses keratinocyte differentiation via protein tyrosine phosphatase non-receptor type 14 (PTPN14) degradation [83, 84]. Activation of yes-associated protein (YAP) transcriptional factor caused by E7-mediated PTPN14 downregulation is crucial for persistence of HPV in basal keratinocytes, in turn contributing to cancer [85]. Although less investigated, E5 and E4 may contribute to viral amplification [86, 87]. E5, a transmembrane protein, cooperates with E7 in cell transformation, proliferation, and suppression of the host immune response. It is also involved in endocytic trafficking and activation of epidermal growth factor receptor-mediated signaling [88]. High-risk HPV E4 protein can induce cell cycle arrest in G2 and stabilizes E2 to support vegetative amplification [89, 90]

In the final stages of the HPV life cycle, *L1* and *L2* transcripts are upregulated via inhibition of early polyadenylation by E2 [91]. L1/L2 structural proteins are formed and the HPV genome is encapsulated in virions that are released during shedding of the upper epithelium. Virus release may be aided by E4 through cytokeratin re-organization [92].

### Persistence of oral HPV

Most oral HPV infections are transient and cleared within 1–2 years without clinical intervention [93–95]. Failure to eliminate HPV triggers persistent infection that lasts from 10 to 30 years [14]. Distribution of oral HPV infection is bimodal and peaks at 30–34 and 60–64 years [23]; the incidence of HPV + OPSCC peaks at 60–64 years [14]. Multiple studies have shown that detection of oral HPV antibodies or DNA years before diagnosis significantly increases the likelihood for development of OPSCC [39, 94, 96]. It is important to note that not all HPV genotypes lead to cancer. HPV16 is currently the leading genotype that contributes to over 80% of HPV + OPSCC [39–41], followed by HPV35 and HPV33 [41, 97, 98]. This differs from the genotype distribution in cervical cancer, where HPV16 and HPV18 are dominant [99]. However, clinical HPV testing typically involves immunohistochemistry for p16, that is limited by its inability to identify specific HPV genotypes [100]. Therefore, the impact of different HPV genotypes in OPSCC remains unclear. Nevertheless, among head and neck cancer subsites, HPV16 is more likely to be distributed at the oropharynx compared to other genotypes such as HPV33 (98, 101). In contrast, non-HPV16 genotypes tend to be found in older patients and contribute to more aggressive tumors, but controversies remain (97, 101*, 102*).

Given the long latency of oral HPV, deciphering the multistep progression between infection to cancer development is important for prevention and early detection of HPV + OPSCC. A few studies have elucidated factors that correlate with oral HPV clearance and persistence; these include male gender, multiple sex partners and oral sex, human immunodeficiency virus (HIV) infection, anti-retroviral therapy, reduced CD4 + T cell count, old age, and smoking (23, 93, 95, 103*). Immunosuppression is likely the key driver of HPV persistence because oral HPV prevalence and increased OPSCC risk occur consistently in immunosuppressed individuals, especially HIV-infected individuals (103*, 104*). HIV/HPV co-infection may provide a permissive immune environment for HPV persistence (105*, 106*), likely due to reduction of CD4+ and CD8 + T lymphocytes (103*, 107*). In a preclinical model, a subset of immunocompetent mice (~30%) injected with HPV16 E6/E7-transformed mouse tonsil keratinocytes cleared the injected HPV+ cells and did not form any spontaneous tumors (107*). However, clearance of HPV+ cells was not observed in mice deficient for B and T lymphocytes (107*). The same study suggests that CD4+ and CD8+ lymphocytes are responsible for the immune response against HPV16 E6/E7 antigens (107*). Together these studies suggest that host immunity is important for viral clearance after HPV infection to prevent carcinogenesis.

Periodontitis is another possible risk factor for oral HPV infection and OPSCC (108*−110*). Periodontitis is a chronic bacterial infection that destroys the periodontium, i.e., the tissues that surround and anchor the teeth (111*). Periodontitis may provide a reservoir for HPV to gain greater access to basal epithelial cells (112*). Furthermore, associated inflammatory responses may promote HPV persistence and carcinogenesis. Tezal et al. reported that patients with HPV+ base-of-tongue tumors have significantly higher alveolar bone loss than HPV(-) base-of-tongue tumors (108*). However, results remain controversial as other studies did not find this correlation (109*). Moreover, the correlation between oral HPV detection and the incidence/severity of periodontal lesions is unclear (110*, 113*, 114*). Overall, longitudinal evaluation may be required to study the link between periodontal inflammation and the persistence of oral HPV. This is especially important because 50–70% of elderly adults above age 50 have periodontitis in the United States (115*), and the economic burden caused by periodontal disease was estimated to be more than US $150 billion in the United States and Europe in 2018 (116*).

In summary, prolonged HPV infection especially in conjunction with immunosuppression, likely drives oropharyngeal carcinogenesis. Given the long latency between HPV infection and OPSCC, follow-up studies extending beyond 10 years could untangle factors associated with viral persistence. Understanding the factors that drive oral persistence and OPSCC development may help to nominate biomarkers that identify individuals at high risk for OPSCC and could act as surrogate markers for premalignant lesions.

## Development, prevention, and treatment strategies for HPV + OPSCC

Prolonged HPV infection increases the expression of E6 and E7 oncoproteins, which modify the epithelium and environment to promote carcinogenesis through multiple mechanisms. Key functions of E6 and E7 oncoproteins include alteration of DNA repair, dysregulation of cell cycle checkpoint, and evasion of immune surveillance (117*−119*). HPV DNA is integrated in host chromosomes in 50–70% of HPV + OPSCC (120*, 121*), suggesting that integration is not an absolute requirement for OPSCC development. Nevertheless, integrated HPV DNA in OPSCC is associated with poor prognosis, although discrepancies exist (32, 122*). The mechanism of integration is unclear but correlates with increased genomic instability of host cells (120*, 123*, 124*). Whether integration events occur before or during carcinogenesis is also elusive.

Among oropharyngeal subsites, the tonsils and base of the tongue have the highest prevalence of HPV infection and cancers [13, 40]. Preferential infection by HPV occurs in tonsilar crypts rather than surface epithelium due to the unique microenvironment of the former (125). The tonsillar crypt is lined by a porous basement membrane that provides HPV with easy access to basal epithelial cells without micro-abrasions (126*, 127*). This also enables immune cells such as lymphocytes and antigen-presenting cells from the surrounding lymphoid follicles to enter the crypt (128*). Interestingly, in the oral cavity, the lymphoid tissue of the tonsil is the first line of defense against foreign pathogens, including HPV (128*). The mechanisms underlying how HPV evades the immune system are still poorly understood, but some studies have proposed that the tonsillar crypt may allow a permissive immune environment for HPV infection due to localized expression of immune checkpoint programmed death ligand-1 (PD-L1) and enabling infiltration of immunosuppressive myeloid populations (125*, 129*, 130*). HPV oncoproteins, such as E5 and E7, also facilitate escape from host immune surveillance by downregulating expression of major histocompatibility complex molecules and inhibiting the cyclic GMP–AMP synthase–stimulator of interferon genes (cGAS-STING) pathway (131*). Furthermore, HPV viruses localize in the biofilm of the tonsillar crypt, providing a reservoir to escape immune surveillance before re-infection (132*).

The growing burden of OPSCC has spurred efforts to encourage HPV vaccination as a primary prevention strategy. In 2020, the United States Food and Drug Administration approved the use of Gardasil 9, a recombinant 9-valent vaccine, in males and females 9 to 45 years to prevent HPV+ cancers including OPSCC. Gardasil 9 induces generation of neutralizing antibodies against HPV L1 protein to prevent primary infection at the cervix and oropharynx. Prophylactic vaccines against HPV16/18 significantly reduce oral HPV prevalence (133*, 134*), but vaccination rates remain low in young adults under the age of 26, especially males (133*). In adolescents between age 13 and 17, only 55–60% were fully vaccinated, far below the national target of 80% (135*). This low vaccination rate is attributed to poor awareness of its benefit, lack of recommendation from healthcare professionals, and limited access to resources especially in underserved and underprivileged regions (136*). Among parents of adolescents, concerns about the safety of the HPV vaccine is often cited as the top reason to decline vaccination for their children (137*). Furthermore, most HPV vaccination programs target females although HPV + OPSCC occurs more frequently in males (14, 138*). Consequently, effects of HPV vaccines will likely be minimal, and the incidence of HPV + OPSCC is expected to continue rising for the next 25 years (139*). An increased awareness of the relationship between HPV vaccination and OPSCC prevention may reverse this trend. In a nationwide study of over 5000 adults in the United States, education on the benefits of HPV vaccines by dental care providers is strongly associated with positive perceptions of this vaccine (140*).

Although currently available HPV vaccines can prevent future infections, they have minimal therapeutic benefits against existing infection and neoplasia (141*−144*). To overcome this limitation, therapeutic vaccines against HPV E6 and/or E7 antigen are under development to potentially treat persistent HPV infections and subvert precancerous and cancerous lesions. (144*−147*). However, most clinical trials that test efficiency of these therapeutic vaccines are performed either in the recurrent/metastatic setting (145*, 146*) or in the definitive setting for locally advanced HPV + OPSCC (144*), especially in conjunction with immune checkpoint inhibitors. Additional investigations are needed to determine the effectiveness of these therapeutic vaccines in early-stage OPSCC.

Given the prevalence of HPV infection and associated carcinogenesis in the tonsils, it is expected that tonsil removal (tonsillectomy) will minimize the potential for HPV infection and malignant transformation (148*). Multiple studies support that tonsillectomy significantly reduced tonsil cancer risk up to 70% compared to individuals with intact tonsils (148*−152*). However, the correlation between prior tonsillectomy and the risk of cancer at other oropharyngeal subsites such as the base of tongue remains inconclusive. While a few studies suggested no association (148*, 149*), others cautioned about a possible elevated risk of carcinoma at the base-of-tongue and/or other subsites of the oropharynx after tonsillectomy (151*−154*). Interestingly, tonsillectomy within 1 year of a tonsil cancer diagnosis was associated with improved survival, suggesting curative potential (148*, 155*, 156*). However, most of these studies did not recommend tonsillectomy as a preventive or curative measure as its associated side effects are unclear (149*, 157*, 158*). There is a limited impact of tonsillectomy on the overall risk of OPSCC, with a possible shift from tonsillar to base-of-tongue cancers, suggesting that the overall malignant capacity of the oropharynx remains the same (151*, 154*).

HPV + OPSCC detected at early stages is treated with single-modality treatment, either surgery (resection of primary tumor with selective neck dissection or radiation therapy (RT) (32, 159*). Transoral robotic surgery (TORS) is widely used as a non-invasive surgical option for early-stage HPV + OPSCC. However, it is important to note that surgery has associated toxicity as suggested by the recently completed ORATOR trial where more early-stage HPV + OPSCC patients undergoing TORS (with or without adjuvant CRT) reported dysphagia than those who underwent definitive radiation therapy as single-modality therapy (160*, 161*). Although TORS and radiation are effective single-modality therapies against early-stage OPSCC, some patients still present adverse features (positive margin and/or extranodal extension), and thus require adjuvant therapy (32, 159*). The majority of OPSCCs are diagnosed at an advanced stage after extensive lymph node involvement that necessitates multimodal treatment, which is associated with adverse side effects [36]. A delay in diagnosis of OPSCC is associated with advanced stage at diagnosis and consequently poorer survival (162*, 163*). Clinicians have highlighted the possibility of reducing treatment intensity, but randomized Phase III trials are needed before de-intensified therapy becomes standard option (159*). Early detection of precancer, including carcinoma-in-situ, will allow diagnosis prior to tumor spread allowing safe delivery of monotherapy with less treatment-associated morbidities while maintaining high curability rates (164*). Furthermore, in-depth information on how HPV infection progresses to precancer is needed to develop therapies that prevent malignant transformation.

Overall, given the limited efficacy of prevention strategies, there is an urgent need to develop screening methods, but challenges remain. In the following sections, we outline the barriers to identifying precancerous lesions in the oropharynx and discuss potential areas for future study.

## Challenges of current technologies to detect premalignant lesions in the oropharynx

The presence of HPV in the oropharynx prior to HPV + OPSCC and long latency from infection to disease onset suggest that premalignant lesions exist. Identification of these lesions is important for early intervention, but none has been clinically or histologically recognized in the oropharynx. In contrast, precancerous lesions are well-characterized at other anatomic sites also infected by HPV such as the cervix, anus, and oral cavity (33, 165*, 166*). This discrepancy could be due to the highly invaginated and complex morphology of the tonsillar crypt, which makes direct visualization of premalignant lesions challenging (167*). Consequently, much of our current knowledge is extrapolated from models of cervical cancer, which differs from OPSCC in biology and clinical progression (168*). There is a need to establish platforms to study how HPV infection in tonsillar crypts and other oropharyngeal sites progresses into viral persistence and cancer.

The lack of defined precancerous lesions in OPSCC means that current diagnostic methods such as physical examinations cannot screen for OPSCC risk (167*). Even for occult early-stage HPV + OPSCC, these diagnostic approaches have limitations (169*). Most HPV + OPSCC are only diagnosed upon the first symptom of disease, usually a neck mass caused by lymph node spread (36, 169*). Previous studies investigated the effectiveness of emerging technologies to diagnose premalignant and sub-clinical lesions, but results are unsatisfactory (170*). Ultrasound and radiological imaging are promising approaches (171*−173*), but their ability to detect precancer and early cancer have not been investigated. Standard white light endoscopy is a mainstay diagnostic approach to determine if clinically suspicious oral lesions need to be biopsied; improvements of this imaging technique will enhance clinical staging and treatment planning. Optical imaging by visualizing autofluorescence (such as Velscope and IllumiScan) or tissue reflectance (such as ViziLite Plus) are commercially available diagnostic adjuncts that allow detection of precursor and early oral cancer lesions, but evidence for diagnosis of oropharyngeal premalignancies is lacking. Even for oral cancer detection, these diagnostic devices may lead to inaccurate diagnosis (174*) and are not recommended for routine evaluation (175*). Narrow band imaging is an emerging optical technique that allows visualization of small and superficial oral lesions by highlighting neoangiogenisis. It has significantly higher diagnostic performance in oral and oropharyngeal cancers compared to traditional white light endoscopy (176*−180*). However, sample sizes of these studies were limited and imaging was often of tumors that were already clinically evident; the diagnostic accuracy of narrow band imaging on clinically occult lesions remains undetermined. Overall, although improvement in imaging techniques has enhanced detection of potentially malignant oral disorders, their effectiveness in precancerous and early OPSCC are uncertain because OPSCCs usually reside within the inner folds of the tonsillar crypt that are not easily visualized on the surface. Research that focuses on designing imaging tools for oropharyngeal premalignant lesions should focus on improvement of visualization of potentially malignant cells hidden in the invaginations of the tonsils and base of tongue.

Cytological screening has been extremely successful as a secondary prevention strategy for cervical cancer. In oral cavity cancer, it is a diagnostic adjunct to detect potentially malignant oral disease and is predicted to have the highest accuracy among all oral cancer diagnosic adjuncts including optical imaging approaches (174*). To evaluate the possibility of cytological testing to diagnose early OPSCC, Fakhry et al. (2011) investigated its effectiveness in two cohorts of individuals with known high risk for OPSCC. The first group comprised those with known oropharyngeal abnormalities, with 70% having confirmed invasive OPSCC, and the second group included HIV-infected patients (170*). Their study showed that cytological assessment reliably detects invasive OPSCC, but is unable to screen for oropharyngeal precancers even in high-risk populations (170*).

Given the limitations of visualization techniques to diagnose a yet to be defined premalignant oropharyngeal lesion, molecular biomarkers that predict malignant changes in the oropharynx may serve as surrogate markers but this will require an understanding of the biology of progression from oral HPV infection to cancer development. The lack of knowledge of the natural history of oral HPV infection means that oral HPV persistence is the only indicator for OPSCC risk. An attractive tool is the detection of oral HPV DNA due to the ease and convenience of collecting oral rinse-and-gargle samples [93]. However, the sensitivity of oral HPV testing varies widely from 30–90% for patients with known and previously untreated HPV + OPSCC (181*−184*). Moreover, many of these studies failed to mimic a screening scenario for individuals with previously undiagnosed OPSCC. In a more recent study, D’Souza et al. (2019) assessed two commercially available oral HPV DNA tests in two cohorts, one comprising HPV + OPSCC patients and matched controls, and the other of non-cancer individuals at high risk of acquiring oral HPV infection or developing HPV + OPSCC (185*). Both oral HPV DNA tests yielded 40–50% specificity in HPV + OPSCC cases compared to healthy controls, while prevalence of oral HPV DNA in the high-risk cohort was low at around 2% (185*). Overall, the low specificity rates of oral HPV DNA tests suggest that many true positives may be missed (185*). Furthermore, most individuals testing positive for oral HPV DNA clear infections within 2 years [94]; absolute risk of OPSCC after detection of oral HPV DNA positivity is extremely low at 0.7% (186*). Therefore, oral HPV DNA testing is not a feasible tool for cancer screening. Subsequent studies explored the potential of methylation markers to detect early OPSCC (187*, 188*). Methylation of *EPB41L3* and HPV16 *L1, L2*, and *E2* in oral gargles was strongly associated with both early and late OPSCC (187*, 188*). However, the specificity for early OPSCC was only 76%, suggesting that additional biomarkers are needed to improve screening especially for early stage OPSCC and precancer (188*).

In contrast, HPV16 E6 antibodies in plasma or serum demonstrate high specificity and sensitivity of over 90% in HPV + OPSCC (189*). Seropositivity of HPV16 E6 has also been detected in cancer-free individuals up to 28 years before diagnosis of OPSCC [96]. Unlike oral HPV DNA that is transiently expressed, HPV16 E6 antibodies are stable and increase with time until OPSCC onset [96]. Individuals with HPV16 E6 seropositivity are 20 to 100 times more likely to develop OPSCC (96, 190*, 191*); notably, HPV16 seropositivity is only weakly associated with other HPV+ cancers, including cancers of the cervix, vagina, vulva, and penis (192*). The 10-year risk of developing OPSCC is predicted to be 20% and 5% for males and females aged 50–65 years, respectively (193*). In contrast, the estimated risk for seronegative individuals is much lower, between 0.01% and 0.25% (193*). These findings were validated by a recent large prospective study where 11 of more than 4000 participants were identified as high-risk for OPSCC based on seropositivity for HPV16 E6 and at least one other early viral antigen (194*). Periodic clinical follow-up of 9 of these 11 high-risk individuals led to successful diagnosis of early-stage HPV + OPSCC in 3 of them within 1–3 years (194*).

Overall, the superior performance of HPV16 E6 antibody makes it a promising candidate surrogate marker for HPV+ oropharyngeal precancer but challenges remain. Despite its rising incidence, HPV + OPSCC is still a rare disease. HPV16 E6 seropositivity exists in less than 1% of healthy individuals (96, 193*) and the majority of HPV16 E6 seropositive individuals will not develop OPSCC (193*, 195*). Screening high-risk populations may be an appropriate cancer prevention strategy, but the criteria for true “high risk” have not been established. Investigation is ongoing to determine whether criteria such as sexual behavior, tobacco exposure, history of HPV-induced malignancies or autoimmune disease, and HIV infection can enrich for individuals who have higher E6 seroprevalence (196*). Another drawback of HPV16 E6 serum antibodies is that they can be detected decades before clinical presentation [96]. However, even if seropositivity is determined, there are no recommended follow-up actions for individuals, causing unnecessary distress. Clinical trials to investigate the effectiveness of current therapeutic interventions are desired.

Another emerging diagnostic biomarker for early OPSCC is HPV circulating tumor DNA (ctDNA). A recent study by Rettig et al. (2022) demonstrated that HPV ctDNA can be detected in individuals up to 43 months before OPSCC diagnosis (197*). Although this study is limited by its small sample size of 10 patients, it highlights the potential for HPV ctDNA as a biomarker for precancer or clinically occult OPSCC (197*). Future studies to validate the utility of HPV ctDNA for early cancer detection in large-scale populations are awaited.

**Fig. 3:**
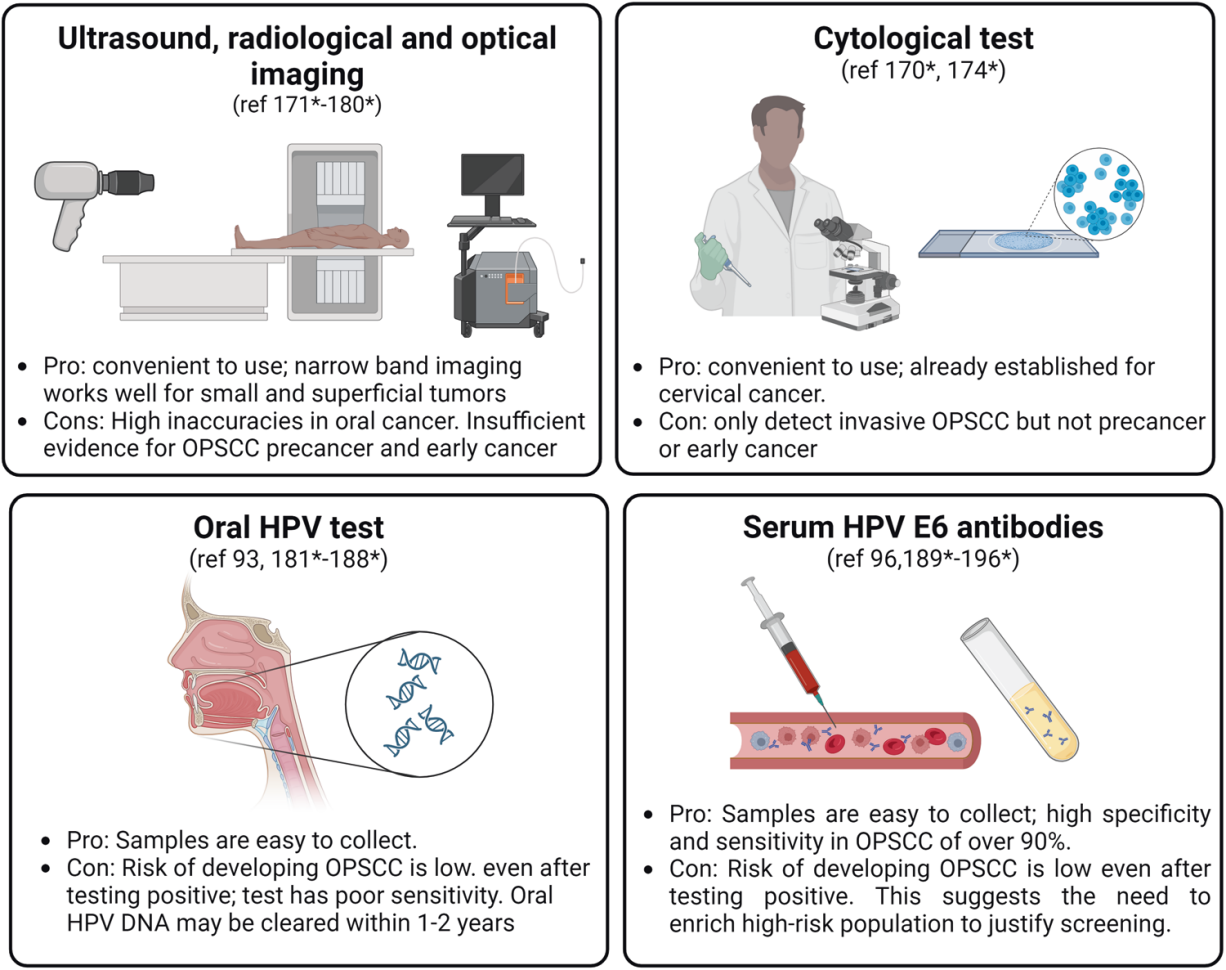
Previous attempts to detect pre-malignant lesions. Created with Biorender.com. References in * are found in Supplementary References.

In summary, attempts to identify biomarkers that predict high risk of OPSCC are sparse, and each nominated biomarker has limitations that impede clinical translation (Fig. 3). While E6 seropositivity is an attractive screen, it is likely that a combination of approaches will be needed to improve screening for OPSCC risk.

## Strategies to decipher progression from HPV infection to OPSCC

### Inferring genetic progression of HPV + OPSCC

The ability to identify genetic events associated with cancer progression may provide valuable information on how normal tissues undergo malignant transformation and enable targeted therapies and cancer prevention. In cancers with clearly defined premalignant lesions such as colorectal cancer (198*), genetic aberrations could be corroborated with distinct clinicopathologic stages to develop a progression timeline. Califano et al. proposed the first genetic progression model for HNSCC (199*), but this was only applicable to HPV(-) HNSCC, which has distinct precancerous lesions. In contrast, HPV + OPSCC does not have a recognized premalignant lesion and lacks the early genetic events identified in HPV(-) HNSCC, such as disruption of *TP53* and *CDKN2A* loci (200*, 201*). It is thus likely that HPV + OPSCC follows a disparate genetic trajectory from its HPV(-) counterpart. There have been several attempts to identify genetic events driving early cancer formation by profiling invasive OPSCC and adjacent non-tumor epithelium (34, 35, 202*). Histologically normal mucosa was more genomically stable than its paired tumor sample, suggesting accumulations in genetic aberrations are needed to form malignant clones [35]. Proposed early driver events include *ZNF750*, *PIK3CA*, and *EP300* mutations [34], but experimental validation is lacking. Immunohistochemical staining of p16 and HPV integration have also been detected in adjacent non-tumor epithelium;[35] it is likely that these events occur before malignant transformation. Interestingly, previous studies on primary human keratinocyte cell lines showed that HPV16 E7 increases *p16*^*INK4A*^ expression [81, 82], confirming p16 as a potential biomarker for precancer. However, although positivity of p16 by immunohistochemistry is a surrogate marker for HPV + OPSCC [17, 26], additional validation studies are needed to evaluate its reliability in predicting precancer.

Although the above-mentioned studies suggest molecular changes from non-tumor epithelium to cancer, the exact timing of genetic driver events in HPV + OPSCC progression is undetermined. Furthermore, it is possible that “histologically normal” epithelium contains occult subclinical lesions. To address this issue, Leshchiner et al. computationally estimated the timing for genetic driver events (203*); gain of chromosome arm 3q and loss of arm 11q were found to be common in HPV + OPSCC and predicted to occur 20 to 30 years before diagnosis (203*). Mutations in *PIK3CA* and *ATM* are expected to drive early HPV+ tumorigenesis (203*). This is consistent with reports that high E6 and E7 expression drives DNA damage and chromosomal instability that drives cancer development (204*, 205*). However, loss of 17p, where the *p53* locus resides, is predicted to happen at the intermediate stage of HPV + OPSCC development (203*). This was unexpected because HPV E6 protein is known to degrade p53 and mutations in *p53* in HPV + OPSCC are rare compared to HPV(-) cancer (206*). Nevertheless, this may suggest an important role for p53 later in HPV + OPSCC development. It is also possible that loss of additional genes in 17p are involved. Another interesting observation from the same study is the timing estimate for initial HPV integration events; these events are predicted to occur more than 25 years before diagnosis of OPSCC, and likely persist throughout tumor development (203*). This coincides with the predicted latency period for HPV infection of around 20–30 years [14], suggesting that HPV integration is an early premalignant event. This is interesting because HPV is integrated in the majority of HPV + OPSCC (50–70%) (120*, 121*). It is unclear whether HPV integration occurs during the viral life cycle before carcinogenesis; experimental studies are needed to validate if integration induces malignant transformation in keratinocytes. In contrast, there is a substantial proportion of patients with unintegrated (episomal) HPV DNA, suggesting that alternative mechanisms may also induce progression from infection to cancer (120*, 121*).

Single-cell RNA sequencing (scRNAseq) analysis may provide insights into the molecular alterations and cell-cell interactions that contribute to progression from premalignancy to OPSCC at single-cell level. In one study, Bedard et al. compared single-cell transcriptomic landscapes between HPV16-infected and uninfected stratified epithelia generated from an immortalized keratinocyte cell line (207*). They identified an undifferentiated keratinocyte subpopulation, termed HIDDEN cells, that was highly amplified in HPV+ epithelium, but absent in HPV(-) epithelium (207*). HIDDEN cells were also detected in alternative models including HPV+ patient-derived tonsillar organoid rafts and in different HPV + OPSCC cohorts, suggesting that these cells are maintained during progression from HPV infection to precancer to cancer (207*). Multiple organoid models demonstrated that HIDDEN cells are mostly enriched in superficial epithelial layers and harbor elevated activity of the ELF3/ESE-1 transcription factor (207*). The HIDDEN cell population was increased in differentiated compared to undifferentiated organotypic models (207*), suggesting that induction or maintenance of this cell subset is dependent on epithelial differentiation. This is consistent with previous studies that suggest that viral oncogene expression increases progressively as infected cells in the basal layer of the tonsillar crypt migrate to more differentiated suprabasal and superficial epithelial layers (32, 208*). Alternative gene signatures of HIDDEN cells include nonconventional differentiation markers such as select mucins and enzymes that regulate o-linked glycosylation, and stem cell markers such as KLF5 and K15 (207*). The HIDDEN subpopulation may be a precursor for HPV + OPSCC, and its associated gene signature suggests that biological processes such as ELF3-induced transcription may trigger malignant transformation. More functional and molecular studies are needed to verify these findings (207*).

In summary, the lack of identifiable premalignant lesions makes it challenging to determine genetic progression of HPV + OPSCC. However, improvements in technology may help to overcome this limitation. A few studies have demonstrated that certain biological events including elevated p16 expression, HPV integration, chromosomal aberrations, and epithelial differentiation occur before clinically identifiable carcinoma. These findings were consistent with previous studies on HPV-induced cancer progression, where the above-mentioned processes were intricately involved. Identifying these molecular changes associated with progression from HPV infection to cancer may help nominate biomarkers to predict early stage OPSCC or precancer. It is also highly possible that the immune microenvironment plays a role in the stepwise progression to cancer, as observed in HPV(-) oral cavity cancer (209*). Future validation should be combined with mechanistic studies to delineate how specific biologic factors promote OPSCC development.

### Novel in vivo models to study the natural history of oral HPV infection

In vivo models of natural HPV infection are essential tools to experimentally investigate progression from infection to persistence to carcinogenesis. Animals including rabbits and dogs were infected with cottontail rabbit papillomavirus (CRPV), rabbit oral papillomavirus (ROPV), canine oral papillomavirus (COPV) or other non-human primate papillomaviruses (210*, 211*). However, many of these species are genetically diverse and not easily maintained or genetically manipulated, making it difficult to investigate the impact of host factors on the viral replication cycle. The discovery of naturally occurring mouse papillomavirus MmuPV provided an opportunity to study infection in laboratory mouse models that are tractable and easily genetically manipulated. A few studies showed that MmuPV infection that primarily occurred in the oral mucosa indirectly triggered secondary lesions at the base of tongue, where OPSCC commonly develops (212*, 213*). However, oral tumors induced from primary infection are different from OPSCC, and it is unclear whether secondary lesions at the base of tongue truly reflect the pathology of human OPSCC. Therefore, precise delivery of the virus at the mouse oropharyngeal region is highly desired, but impeded by technical challenges. Firstly, deliberate infection to the oropharynx requires anesthesia, but the duration of anesthesia is difficult to control. Secondly, the virus must be delivered to the basal cell layer of the pharynx, but accurate manipulation of the needle in this region is challenging and may cause unnecessary injury to the epithelium. In a recent study, Bilger et al. attempted to overcome these challenges by developing a novel tool for efficient nasal anesthesia and precise MmuPV infection at the oropharynx (214*). Unlike humans, histologically distinct stages of premalignant lesions could be observed in the oropharyngeal epithelium of infected mice (213*, 214*); likely due to the lack of a highly invaginated tonsillar crypt in mice, making precancerous lesions easily visible (213*, 214*). However, it is unclear if virus-induced lesions in mice fully recapitulate HPV-induced OPSCC pathogenesis in humans. Nevertheless, these MmuPV-induced models are useful tools to reveal insights on progression from viral infection to cancer in the oropharynx.

Although infection-based models have the key advantage of providing differential stages from non-infection to viral persistence and malignancy, not all infected mice develop tumors (213*). In contrast, genetically modified murine models (GEMMs) enable higher efficiency of viral-induced carcinogenesis and are important tools for investigations of cancer progression (215*). However, most currently available GEMMs for HNSCC are designed to model HPV(-) oral SCC (216*). In contrast, GEMMs for HPV + HNSCC are sparse and previous attempts led to nonspecific targeting at the cutaneous epithelium, while others target the oral cavity, which is not the predominant site for HPV infection (216*). Other caveats of previous GEMM models for HPV + HNSCC are: (i) require concurrent mutations in genes such as *NRAS* that are infrequently involved in OPSCC; and (ii) are limited to immunocompromised mice, making investigations of the immune response to HPV+ tumors challenging (216). In 2019, Carper et al. overcame these limitations and reported the first conditional knock-in GEMM that allows post-natal expression of E6 and E7 oncogenes specifically in the oropharyngeal epithelium (217*). Co-expression of a *PIK3CA*^E545k^ mutant was introduced in this GEMM to induce OPSCC development (217) this is because mutations in PIK3CA, particularly E542K and E545K occur frequently in HPV + OPSCC (206*, 218*, 219*). Precancerous lesions were observed in the murine oropharynx (217*). Thus, early-stage lesions could also be studied in this model. However, given that progressive stages of epithelial dysplasia observed in mice do not appear in human OPSCC, it is unclear whether cancer progression in this GEMM fully recapitulates that in humans. The authors also reported that most OPSCC in their murine models were in situ carcinoma, although regional metastasis occurred (217*). Furthermore, the GEMM was immunocompetent, allowing the authors to study the role of immunity in OPSCC development (217*). Another caveat of GEMMs is that they require either chemical or genetic manipulation, which may not mimic pathogenesis in humans; selection of appropriate models is critical. Development and refinement of murine models that recapitulate key features of HPV + OPSCC, are needed to explore the biology and therapy.

**Fig. 4:**
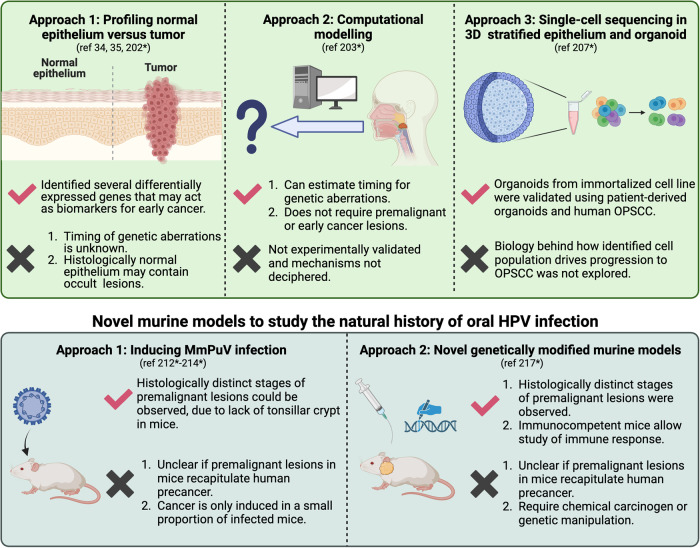
Previous attempts to detect pre-malignant lesions. Created with Biorender.com. References in * are found in Supplementary References.

Overall, understanding how HPV infection progresses to OPSCC is important but hindered because intermediate precancerous stages have not been identified. Recent studies have attempted to overcome this limitation by (i) profiling histologically normal oropharynx, (ii) computationally estimating timings of genetic aberrations, and (iii) developing in vivo models to recapitulate oral HPV infection, persistence, and OPSCC development (Fig. 4).

## Conclusion and future directions

Given the growing burden of HPV + OPSCC, prevention is a high priority. HPV vaccines are ideal as primary prevention strategies, but vaccination rates are low (220*), and the impact of these vaccines is likely limited for the next two decades (139*, 220*). Therefore, cancer screening would enable timely intervention to decrease treatment-associated morbidity and mortality. Unfortunately, current technologies are inadequate for diagnosing precancer and early cancer. HPV16 E6 serum antibody is the most promising biomarker with high positive predictive value, but alone may be insufficient for accurate prediction (194*). It is possible that a combination of screening strategies is required for accurate detection. Future studies should continue to identify biomarkers and determine the combination that most reliably detects HPV persistence and oropharyngeal precancer. Several challenges remain; it is unclear when and whether all persistent HPV infections will ultimately transform to cancer. Therapeutic HPV vaccines under development may benefit individuals with confirmed persistent HPV infection but clinical trials are needed to test this hypothesis. Furthermore, even if precancer is detected, it is important to confirm if identification merits a change in intervention for patients. Although TORS and definitive radiation therapy are highly effective for patients who have stage I–II OPSCC with no observed high-risk pathologic features, their effectiveness in oropharyngeal precancer has not been investigated. Given the long latency between viral infection and OPSCC development, determining stages of progression of precancer to cancer will help to identify biomarkers and enable intervention strategies based on the likelihood of progression.

### Supplementary information

Supplementary References
