# Supplementary material for: HPV-associated oropharyngeal cancer: in search of surrogate biomarkers for early lesions
Source: Oncogene. 2024 Jan 8;43(8):543–54. doi: 10.1038/s41388-023-02927-9 (PMC10873204; doi:10.1038/s41388-023-02927-9)
Supplement: Supplementary file 1 — Supplementary References [file 41388_2023_2927_MOESM1_ESM.docx]

**Supplementary References**

**The following references are cited in the manuscript (text and tables) followed by an asterisk (*) and are not included in the list of references of the main text.**

101. Chatfield-Reed K, Gui S, O'Neill WQ, Teknos TN, Pan Q. HPV33+ HNSCC is associated with poor prognosis and has unique genomic and immunologic landscapes. Oral Oncology. 2020;100:104488.

102. Varier I, Keeley BR, Krupar R, Patsias A, Dong J, Gupta N, et al. Clinical characteristics and outcomes of oropharyngeal carcinoma related to high-risk non–human papillomavirus16 viral subtypes. Head & Neck. 2016;38(9):1330-7.

103. Riddell J, Brouwer AF, Walline HM, Campredon LP, Meza R, Eisenberg MC, et al. Oral human papillomavirus prevalence, persistence, and risk-factors in HIV-positive and HIV-negative adults. Tumour Virus Research. 2022;13:200237.

104. Parisi SG, Cruciani M, Scaggiante R, Boldrin C, Andreis S, Bello FD, et al. Anal and oral human papillomavirus (HPV) infection in HIV-infected subjects in northern Italy: a longitudinal cohort study among men who have sex with men. BMC Infectious Diseases. 2011;11(1):150.

105. Verma M, Erwin S, Abedi V, Hontecillas R, Hoops S, Leber A, et al. Modeling the Mechanisms by Which HIV-Associated Immunosuppression Influences HPV Persistence at the Oral Mucosa. PLOS ONE. 2017;12(1):e0168133.

106. Tugizov SM, Herrera R, Chin-Hong P, Veluppillai P, Greenspan D, Michael Berry J, et al. HIV-associated disruption of mucosal epithelium facilitates paracellular penetration by human papillomavirus. Virology. 2013;446(1):378-88.

107. Williams R, Lee DW, Elzey BD, Anderson ME, Hostager BS, Lee JH. Preclinical models of HPV+ and HPV− HNSCC in mice: An immune clearance of HPV+ HNSCC. Head & Neck. 2009;31(7):911-8.

108. Tezal M, Sullivan Nasca M, Stoler DL, Melendy T, Hyland A, Smaldino PJ, et al. Chronic Periodontitis−Human Papillomavirus Synergy in Base of Tongue Cancers. Archives of Otolaryngology–Head & Neck Surgery. 2009;135(4):391-6.

109. Ortiz AP, Ramos-Cartagena JM, García-Camacho SI, Andriankaja OM, Pérez CM. Is Human Papilloma Virus Infection Linked to Periodontitis? A Narrative Review. Current Oral Health Reports. 2019;6(1):22-30.

110. Ortiz AP, Ramos-Cartagena JM, García-Camacho SI, Andriankaja OM, Pérez CM. Is Human Papilloma Virus Infection Linked to Periodontitis? A Narrative Review. Curr Oral Health Rep. 2019;6(1):22-30.

111. Kinane DF, Stathopoulou PG, Papapanou PN. Periodontal diseases. Nature Reviews Disease Primers. 2017;3(1):17038.

112. Hormia M, Willberg J, Ruokonen H, Syrjänen S. Marginal Periodontium as a Potential Reservoir of Human Papillomavirus in Oral Mucosa. Journal of Periodontology. 2005;76(3):358-63.

113. Fuster-Rossello L, Ribotta E, Cuffini C, Fuster-Juan M. Human papilloma virus in oral mucosa and its association with periodontal status of gynecologically infected women. Acta Odontol Latinoam. 2014;27(2):82-8.

114. Ortiz AP, González D, Vivaldi-Oliver J, Castañeda M, Rivera V, Díaz E, et al. Periodontitis and oral human papillomavirus infection among Hispanic adults. Papillomavirus Research. 2018;5:128-33.

115. Eke PI, Dye BA, Wei L, Slade GD, Thornton-Evans GO, Borgnakke WS, et al. Update on Prevalence of Periodontitis in Adults in the United States: NHANES 2009 to 2012. Journal of Periodontology. 2015;86(5):611-22.

116. Botelho J, Machado V, Leira Y, Proença L, Chambrone L, Mendes JJ. Economic burden of periodontitis in the United States and Europe: An updated estimation. Journal of Periodontology. 2022;93(3):373-9.

117. Lechner M, Liu J, Masterson L, Fenton TR. HPV-associated oropharyngeal cancer: epidemiology, molecular biology and clinical management. Nature Reviews Clinical Oncology. 2022;19(5):306-27.

118. Della Fera AN, Warburton A, Coursey TL, Khurana S, McBride AA. Persistent Human Papillomavirus Infection. Viruses. 2021;13(2).

119. Mittal S, Banks L. Molecular mechanisms underlying human papillomavirus E6 and E7 oncoprotein-induced cell transformation. Mutation Research/Reviews in Mutation Research. 2017;772:23-35.

120. Parfenov M, Pedamallu CS, Gehlenborg N, Freeman SS, Danilova L, Bristow CA, et al. Characterization of HPV and host genome interactions in primary head and neck cancers. Proceedings of the National Academy of Sciences. 2014;111(43):15544-9.

121. Koneva LA, Zhang Y, Virani S, Hall PB, McHugh JB, Chepeha DB, et al. HPV Integration in HNSCC Correlates with Survival Outcomes, Immune Response Signatures, and Candidate Drivers. Molecular Cancer Research. 2018;16(1):90-102.

122. Balaji H, Demers I, Wuerdemann N, Schrijnder J, Kremer B, Klussmann JP, et al. Causes and Consequences of HPV Integration in Head and Neck Squamous Cell Carcinomas: State of the Art. Cancers. 2021;13(16).

123. Labarge B, Hennessy M, Zhang L, Goldrich D, Chartrand S, Purnell C, et al. Human Papillomavirus Integration Strictly Correlates with Global Genome Instability in Head and Neck Cancer. Molecular Cancer Research. 2022;20(9):1420-8.

124. Symer DE, Akagi K, Geiger HM, Song Y, Li G, Emde A-K, et al. Diverse tumorigenic consequences of human papillomavirus integration in primary oropharyngeal cancers. Genome Research. 2022;32(1):55-70.

125. Mattox AK, Roelands J, Saal TM, Cheng Y, Rinchai D, Hendrickx W, et al. Myeloid Cells Are Enriched in Tonsillar Crypts, Providing Insight into the Viral Tropism of Human Papillomavirus. The American Journal of Pathology. 2021;191(10):1774-86.

126. Perry ME. The specialised structure of crypt epithelium in the human palatine tonsil and its functional significance. J Anat. 1994;185 ( Pt 1)(Pt 1):111-27.

127. Roberts S, Evans D, Mehanna H, Parish JL. Modelling human papillomavirus biology in oropharyngeal keratinocytes. Philosophical Transactions of the Royal Society B: Biological Sciences. 2019;374(1773):20180289.

128. Ferris RL, Westra W. Oropharyngeal Carcinoma with a Special Focus on HPV-Related Squamous Cell Carcinoma. Annual Review of Pathology: Mechanisms of Disease. 2023;18(1):515-35.

129. Galliverti G, Wullschleger S, Tichet M, Murugan D, Zangger N, Horton W, et al. Myeloid Cells Orchestrate Systemic Immunosuppression, Impairing the Efficacy of Immunotherapy against HPV+ Cancers. Cancer Immunology Research. 2020;8(1):131-45.

130. Lyford-Pike S, Peng S, Young GD, Taube JM, Westra WH, Akpeng B, et al. Evidence for a Role of the PD-1:PD-L1 Pathway in Immune Resistance of HPV-Associated Head and Neck Squamous Cell Carcinoma. Cancer Research. 2013;73(6):1733-41.

131. Shamseddine AA, Burman B, Lee NY, Zamarin D, Riaz N. Tumor Immunity and Immunotherapy for HPV-Related Cancers. Cancer Discovery. 2021;11(8):1896-912.

132. Rieth KKS, Gill SR, Lott-Limbach AA, Merkley MA, Botero N, Allen PD, et al. Prevalence of High-Risk Human Papillomavirus in Tonsil Tissue in Healthy Adults and Colocalization in Biofilm of Tonsillar Crypts. JAMA Otolaryngology–Head & Neck Surgery. 2018;144(3):231-7.

133. Chaturvedi AK, Graubard BI, Broutian T, Pickard RKL, Tong Z-Y, Xiao W, et al. Effect of Prophylactic Human Papillomavirus (HPV) Vaccination on Oral HPV Infections Among Young Adults in the United States. Journal of Clinical Oncology. 2018;36(3):262-7.

134. Hirth JM, Chang M, Resto VA, Guo F, Berenson AB. Prevalence of oral human papillomavirus by vaccination status among young adults (18–30years old). Vaccine. 2017;35(27):3446-51.

135. Pingali C, Yankey D, Elam-Evans LD, Markowitz LE, Valier MR, Fredua B, et al. National Vaccination Coverage Among Adolescents Aged 13-17 Years - National Immunization Survey-Teen, United States, 2021. MMWR Morb Mortal Wkly Rep. 2022;71(35):1101-8.

136. Holman DM, Benard V, Roland KB, Watson M, Liddon N, Stokley S. Barriers to Human Papillomavirus Vaccination Among US Adolescents: A Systematic Review of the Literature. JAMA Pediatrics. 2014;168(1):76-82.

137. Adjei Boakye E, Nair M, Abouelella DK, Joseph CLM, Gerend MA, Subramaniam DS, et al. Trends in Reasons for Human Papillomavirus Vaccine Hesitancy: 2010–2020. Pediatrics. 2023;151(6).

138. Guo F, Chang M, Scholl M, McKinnon B, Berenson AB. Trends in Oropharyngeal Cancer Incidence Among Adult Men and Women in the United States From 2001 to 2018. Frontiers in Oncology. 2022;12.

139. Zhang Y, Fakhry C, D’Souza G. Projected Association of Human Papillomavirus Vaccination With Oropharynx Cancer Incidence in the US, 2020-2045. JAMA Oncology. 2021;7(10):e212907-e.

140. Naavaal S, Demopoulos CA, Kelly A, Tranby E, Frantsve-Hawley J. Perceptions about human papillomavirus vaccine and oropharyngeal cancers, and the role of dental care providers in human papillomavirus prevention among US adults. The Journal of the American Dental Association. 2023;154(4):321-9.

141. Karimi-Zarchi M, Allahqoli L, Nehmati A, Kashi AM, Taghipour-Zahir S, Alkatout I. Can the prophylactic quadrivalent HPV vaccine be used as a therapeutic agent in women with CIN? A randomized trial. BMC Public Health. 2020;20(1):274.

142. Lichter K, Krause D, Xu J, Tsai SHL, Hage C, Weston E, et al. Adjuvant Human Papillomavirus Vaccine to Reduce Recurrent Cervical Dysplasia in Unvaccinated Women: A Systematic Review and Meta-analysis. Obstetrics & Gynecology. 2020;135(5).

143. Stankiewicz Karita HC, Hauge K, Magaret A, Mao C, Schouten J, Grieco V, et al. Effect of Human Papillomavirus Vaccine to Interrupt Recurrence of Vulvar and Anal Neoplasia (VIVA): A Trial Protocol. JAMA Network Open. 2019;2(4):e190819-e.

144. Morand GB, Cardona I, Cruz SBSC, Mlynarek AM, Hier MP, Alaoui-Jamali MA, et al. Therapeutic Vaccines for HPV-Associated Oropharyngeal and Cervical Cancer: The Next De-Intensification Strategy? International Journal of Molecular Sciences. 2022;23(15):8395.

145. Aggarwal C, Cohen RB, Morrow MP, Kraynyak KA, Sylvester AJ, Knoblock DM, et al. Immunotherapy Targeting HPV16/18 Generates Potent Immune Responses in HPV-Associated Head and Neck Cancer. Clinical Cancer Research. 2019;25(1):110-24.

146. Aggarwal C, Saba NF, Algazi AP, Sukari A, Seiwert T, Haigentz M, et al. 916MO Safety and efficacy of MEDI0457 plus durvalumab in patients (pts) with human papillomavirus-associated recurrent/metastatic head and neck squamous cell carcinoma (HPV+ R/M HNSCC). Annals of Oncology. 2020;31:S661-S2.

147. Grunwitz C, Salomon N, Vascotto F, Selmi A, Bukur T, Diken M, et al. HPV16 RNA-LPX vaccine mediates complete regression of aggressively growing HPV-positive mouse tumors and establishes protective T cell memory. OncoImmunology. 2019;8(9):e1629259.

148. Fakhry C, Andersen KK, Christensen J, Agrawal N, Eisele DW. The Impact of Tonsillectomy upon the Risk of Oropharyngeal Carcinoma Diagnosis and Prognosis in the Danish Cancer Registry. Cancer Prevention Research. 2015;8(7):583-9.

149. Chaturvedi AK, Song H, Rosenberg PS, Ramqvist T, Anderson WF, Munck-Wikland E, et al. Tonsillectomy and Incidence of Oropharyngeal Cancers. Cancer Epidemiology, Biomarkers & Prevention. 2016;25(6):944-50.

150. Achim M F, Jochen P W. Tonsillectomy as a Prevention Strategy in the Light of Increasing Incidence of Oropharyngeal Cancer – a Research of Current Literature. Anticancer Research. 2021;41(3):1157.

151. Altenhofen B, DeWees TA, Ahn JW, Yeat NC, Goddu S, Chen I, et al. Childhood tonsillectomy alters the primary distribution of HPV-related oropharyngeal squamous cell carcinoma. Laryngoscope Investigative Otolaryngology. 2020;5(2):210-6.

152. Alharbi H, Saleh W, Yue S, Fernandes RP. Association between tonsillectomy and oropharyngeal cancer risk: a retrospective cohort study. Oral and Maxillofacial Surgery. 2023.

153. Zevallos JP, Mazul AL, Rodriguez N, Weissler MC, Brennan P, Anantharaman D, et al. Previous tonsillectomy modifies odds of tonsil and base of tongue cancer. British Journal of Cancer. 2016;114(7):832-8.

154. Combes J-D, Voisin N, Périé S, Malard O, Jegoux F, Nadjingar R, et al. History of tonsillectomy and risk of oropharyngeal cancer. Oral Oncology. 2021;117:105302.

155. Holliday MA, Tavaluc R, Zhuang T, Wang H, Davidson B. Oncologic Benefit of Tonsillectomy in Stage I and II Tonsil Cancer: A Surveillance Epidemiology and End Results Database Review. JAMA Otolaryngology–Head & Neck Surgery. 2013;139(4):362-6.

156. Yildirim G, Morrison WH, Rosenthal DI, Sturgis EM, Papadimitrakopoulou VA, Schwartz DL, et al. Outcomes of patients with tonsillar carcinoma treated with post-tonsillectomy radiation therapy. Head & Neck. 2010;32(4):473-80.

157. Misiukiewicz K, Posner M. Role of Prophylactic Bilateral Tonsillectomy as a Cancer Preventive Strategy. Cancer Prevention Research. 2015;8(7):580-2.

158. Weinstein GS, O’Malley BW, Jr, Snyder W, Sherman E, Quon H. Transoral Robotic Surgery: Radical Tonsillectomy. Archives of Otolaryngology–Head & Neck Surgery. 2007;133(12):1220-6.

159. Caudell JJ, Gillison ML, Maghami E, Spencer S, Pfister DG, Adkins D, et al. NCCN Guidelines® Insights: Head and Neck Cancers, Version 1.2022: Featured Updates to the NCCN Guidelines. Journal of the National Comprehensive Cancer Network. 2022;20(3):224-34.

160. Nichols AC, Theurer J, Prisman E, Read N, Berthelet E, Tran E, et al. Radiotherapy versus transoral robotic surgery and neck dissection for oropharyngeal squamous cell carcinoma (ORATOR): an open-label, phase 2, randomised trial. The Lancet Oncology. 2019;20(10):1349-59.

161. Palma DA, Prisman E, Berthelet E, Tran E, Hamilton S, Wu J, et al. Assessment of Toxic Effects and Survival in Treatment Deescalation With Radiotherapy vs Transoral Surgery for HPV-Associated Oropharyngeal Squamous Cell Carcinoma: The ORATOR2 Phase 2 Randomized Clinical Trial. JAMA Oncology. 2022;8(6):845-51.

162. Seoane J, Takkouche B, Varela-Centelles P, Tomás I, Seoane-Romero JM. Impact of delay in diagnosis on survival to head and neck carcinomas: a systematic review with meta-analysis. Clinical Otolaryngology. 2012;37(2):99-106.

163. Pitchers M, Martin C. Delay in referral of oropharyngeal squamous cell carcinoma to secondary care correlates with a more advanced stage at presentation, and is associated with poorer survival. British Journal of Cancer. 2006;94(7):955-8.

164. Eisbruch A, Harris J, Garden AS, Chao CKS, Straube W, Harari PM, et al. Multi-Institutional Trial of Accelerated Hypofractionated Intensity-Modulated Radiation Therapy for Early-Stage Oropharyngeal Cancer (RTOG 00-22). International Journal of Radiation Oncology*Biology*Physics. 2010;76(5):1333-8.

165. Steenbergen RDM, Snijders PJF, Heideman DAM, Meijer CJLM. Clinical implications of (epi)genetic changes in HPV-induced cervical precancerous lesions. Nature Reviews Cancer. 2014;14(6):395-405.

166. Lerman MA, Almazrooa S, Lindeman N, Hall D, Villa A, Woo S-B. HPV-16 in a distinct subset of oral epithelial dysplasia. Modern Pathology. 2017;30(12):1646-54.

167. Timbang MR, Sim MW, Bewley AF, Farwell DG, Mantravadi A, Moore MG. HPV-related oropharyngeal cancer: a review on burden of the disease and opportunities for prevention and early detection. Human Vaccines & Immunotherapeutics. 2019;15(7-8):1920-8.

168. Berman TA, Schiller JT. Human papillomavirus in cervical cancer and oropharyngeal cancer: One cause, two diseases. Cancer. 2017;123(12):2219-29.

169. O'Sullivan B, Huang SH, Su J, Garden AS, Sturgis EM, Dahlstrom K, et al. Development and validation of a staging system for HPV-related oropharyngeal cancer by the International Collaboration on Oropharyngeal cancer Network for Staging (ICON-S): a multicentre cohort study. The Lancet Oncology. 2016;17(4):440-51.

170. Fakhry C, Rosenthal BT, Clark DP, Gillison ML. Associations between Oral HPV16 Infection and Cytopathology: Evaluation of an Oropharyngeal “Pap-Test Equivalent” in High-Risk Populations. Cancer Prevention Research. 2011;4(9):1378-84.

171. Lang Kuhs KA, Wood CB, Wiggleton J, Aulino JM, Latimer B, Smith DK, et al. Transcervical sonography and human papillomavirus 16 E6 antibodies are sensitive for the detection of oropharyngeal cancer. Cancer. 2020;126(11):2658-65.

172. Coquia SF, Hamper UM, Holman ME, DeJong MR, Subramaniam RM, Aygun N, et al. Visualization of the Oropharynx With Transcervical Ultrasound. American Journal of Roentgenology. 2015;205(6):1288-94.

173. Salzillo TC, Taku N, Wahid KA, McDonald BA, Wang J, van Dijk LV, et al. Advances in Imaging for HPV-Related Oropharyngeal Cancer: Applications to Radiation Oncology. Seminars in Radiation Oncology. 2021;31(4):371-88.

174. Lingen MW, Tampi MP, Urquhart O, Abt E, Agrawal N, Chaturvedi AK, et al. Adjuncts for the evaluation of potentially malignant disorders in the oral cavity: Diagnostic test accuracy systematic review and meta-analysis—a report of the American Dental Association. The Journal of the American Dental Association. 2017;148(11):797-813.e52.

175. Lingen MW, Abt E, Agrawal N, Chaturvedi AK, Cohen E, D’Souza G, et al. Evidence-based clinical practice guideline for the evaluation of potentially malignant disorders in the oral cavity: A report of the American Dental Association. The Journal of the American Dental Association. 2017;148(10):712-27.e10.

176. Piazza C, Del Bon F, Paderno A, Grazioli P, Perotti P, Barbieri D, et al. The diagnostic value of narrow band imaging in different oral and oropharyngeal subsites. European Archives of Oto-Rhino-Laryngology. 2016;273(10):3347-53.

177. van Schaik JE, Halmos GB, Witjes MJH, Plaat BEC. An overview of the current clinical status of optical imaging in head and neck cancer with a focus on Narrow Band imaging and fluorescence optical imaging. Oral Oncology. 2021;121:105504.

178. Lin Y-C, Watanabe A, Chen W-C, Lee K-F, Lee I-L, Wang W-H. Narrowband Imaging for Early Detection of Malignant Tumors and Radiation Effect After Treatment of Head and Neck Cancer. Archives of Otolaryngology–Head & Neck Surgery. 2010;136(3):234-9.

179. Muto M, Nakane M, Katada C, Sano Y, Ohtsu A, Esumi H, et al. Squamous cell carcinoma in situ at oropharyngeal and hypopharyngeal mucosal sites. Cancer. 2004;101(6):1375-81.

180. Muto M, Minashi K, Yano T, Saito Y, Oda I, Nonaka S, et al. Early Detection of Superficial Squamous Cell Carcinoma in the Head and Neck Region and Esophagus by Narrow Band Imaging: A Multicenter Randomized Controlled Trial. Journal of Clinical Oncology. 2010;28(9):1566-72.

181. Agrawal Y, Koch WM, Xiao W, Westra WH, Trivett AL, Symer DE, et al. Oral Human Papillomavirus Infection Before and After Treatment for Human Papillomavirus 16–Positive and Human Papillomavirus 16–Negative Head and Neck Squamous Cell Carcinoma. Clinical Cancer Research. 2008;14(21):7143-50.

182. Rettig EM, Wentz A, Posner MR, Gross ND, Haddad RI, Gillison ML, et al. Prognostic Implication of Persistent Human Papillomavirus Type 16 DNA Detection in Oral Rinses for Human Papillomavirus–Related Oropharyngeal Carcinoma. JAMA Oncology. 2015;1(7):907-15.

183. Chai RC, Lim Y, Frazer IH, Wan Y, Perry C, Jones L, et al. A pilot study to compare the detection of HPV-16 biomarkers in salivary oral rinses with tumour p16INK4a expression in head and neck squamous cell carcinoma patients. BMC Cancer. 2016;16(1):178.

184. Zhao M, Rosenbaum E, Carvalho AL, Koch W, Jiang W, Sidransky D, et al. Feasibility of quantitative PCR-based saliva rinse screening of HPV for head and neck cancer. International Journal of Cancer. 2005;117(4):605-10.

185. D'Souza G, Clemens G, Troy T, Castillo RG, Struijk L, Waterboer T, et al. Evaluating the Utility and Prevalence of HPV Biomarkers in Oral Rinses and Serology for HPV-related Oropharyngeal Cancer. Cancer Prevention Research. 2019;12(10):689-700.

186. D’Souza G, McNeel TS, Fakhry C. Understanding personal risk of oropharyngeal cancer: risk-groups for oncogenic oral HPV infection and oropharyngeal cancer. Annals of Oncology. 2017;28(12):3065-9.

187. Giuliano AR, Nedjai B, Lorincz AT, Schell MJ, Rahman S, Banwait R, et al. Methylation of HPV 16 and EPB41L3 in oral gargles: Associations with oropharyngeal cancer detection and tumor characteristics. International Journal of Cancer. 2020;146(4):1018-30.

188. Dickey BL, Nedjai B, Preece MD, Schell MJ, Boulware D, Whiting J, et al. Methylation of HPV16 and EPB41L3 in oral gargles and the detection of early and late oropharyngeal cancer. Cancer Medicine. 2022;11(20):3735-42.

189. Holzinger D, Wichmann G, Baboci L, Michel A, Höfler D, Wiesenfarth M, et al. Sensitivity and specificity of antibodies against HPV16 E6 and other early proteins for the detection of HPV16-driven oropharyngeal squamous cell carcinoma. International Journal of Cancer. 2017;140(12):2748-57.

190. Kreimer AR, Johansson M, Waterboer T, Kaaks R, Chang-Claude J, Drogen D, et al. Evaluation of Human Papillomavirus Antibodies and Risk of Subsequent Head and Neck Cancer. Journal of Clinical Oncology. 2013;31(21):2708-15.

191. Kreimer AR, Johansson M, Yanik EL, Katki HA, Check DP, Lang Kuhs KA, et al. Kinetics of the Human Papillomavirus Type 16 E6 Antibody Response Prior to Oropharyngeal Cancer. JNCI: Journal of the National Cancer Institute. 2017;109(8).

192. Kreimer AR, Brennan P, Lang Kuhs KA, Waterboer T, Clifford G, Franceschi S, et al. Human Papillomavirus Antibodies and Future Risk of Anogenital Cancer: A Nested Case-Control Study in the European Prospective Investigation Into Cancer and Nutrition Study. Journal of Clinical Oncology. 2015;33(8):877-84.

193. Robbins HA, Ferreiro-Iglesias A, Waterboer T, Brenner N, Nygard M, Bender N, et al. Absolute Risk of Oropharyngeal Cancer After an HPV16-E6 Serology Test and Potential Implications for Screening: Results From the Human Papillomavirus Cancer Cohort Consortium. Journal of Clinical Oncology. 2022;40(31):3613-22.

194. Busch C-J, Hoffmann AS, Viarisio D, Becker BT, Rieckmann T, Betz C, et al. Detection of stage I HPV-driven oropharyngeal cancer in asymptomatic individuals in the Hamburg City Health Study using HPV16 E6 serology &#x2013; A proof-of-concept study. eClinicalMedicine. 2022;53.

195. Brenner N, Mentzer AJ, Hill M, Almond R, Allen N, Pawlita M, et al. Characterization of human papillomavirus (HPV) 16 E6 seropositive individuals without HPV-associated malignancies after 10 years of follow-up in the UK Biobank. eBioMedicine. 2020;62.

196. D’Souza G, Tewari SR, Troy T, Waterboer T, Struijk L, Castillo R, et al. Prevalence of oral and blood oncogenic human papillomavirus biomarkers among an enriched screening population: Baseline results of the MOUTH study. Cancer. 2023;129(15):2373-84.

197. Rettig EM, Faden DL, Sandhu S, Wong K, Faquin WC, Warinner C, et al. Detection of circulating tumor human papillomavirus DNA before diagnosis of HPV-positive head and neck cancer. International Journal of Cancer. 2022;151(7):1081-5.

198. Vogelstein B, Fearon ER, Hamilton SR, Kern SE, Preisinger AC, Leppert M, et al. Genetic Alterations during Colorectal-Tumor Development. New England Journal of Medicine. 1988;319(9):525-32.

199. Califano J, van der Riet P, Westra W, Nawroz H, Clayman G, Piantadosi S, et al. Genetic Progression Model for Head and Neck Cancer: Implications for Field Cancerization1. Cancer Research. 1996;56(11):2488-92.

200. Seiwert TY, Zuo Z, Keck MK, Khattri A, Pedamallu CS, Stricker T, et al. Integrative and Comparative Genomic Analysis of HPV-Positive and HPV-Negative Head and Neck Squamous Cell Carcinomas. Clinical Cancer Research. 2015;21(3):632-41.

201. Chung CH, Guthrie VB, Masica DL, Tokheim C, Kang H, Richmon J, et al. Genomic alterations in head and neck squamous cell carcinoma determined by cancer gene-targeted sequencing. Annals of Oncology. 2015;26(6):1216-23.

202. Masterson L, Sorgeloos F, Winder D, Lechner M, Marker A, Malhotra S, et al. Deregulation of SYCP2 predicts early stage human papillomavirus-positive oropharyngeal carcinoma: A prospective whole transcriptome analysis. Cancer Science. 2015;106(11):1568-75.

203. Leshchiner I, Mroz EA, Cha J, Rosebrock D, Spiro O, Bonilla-Velez J, et al. Inferring early genetic progression in cancers with unobtainable premalignant disease. Nature Cancer. 2023;4(4):550-63.

204. Solinas-Toldo S, Dürst M, Lichter P. Specific chromosomal imbalances in human papillomavirus-transfected cells during progression toward immortality. Proceedings of the National Academy of Sciences. 1997;94(8):3854-9.

205. Duensing S, Lee LY, Duensing A, Basile J, Piboonniyom S-o, Gonzalez S, et al. The human papillomavirus type 16 E6 and E7 oncoproteins cooperate to induce mitotic defects and genomic instability by uncoupling centrosome duplication from the cell division cycle. Proceedings of the National Academy of Sciences. 2000;97(18):10002-7.

206. Lawrence MS, Sougnez C, Lichtenstein L, Cibulskis K, Lander E, Gabriel SB, et al. Comprehensive genomic characterization of head and neck squamous cell carcinomas. Nature. 2015;517(7536):576-82.

207. Bedard MC, Chihanga T, Carlile A, Jackson R, Brusadelli MG, Lee D, et al. Single cell transcriptomic analysis of HPV16-infected epithelium identifies a keratinocyte subpopulation implicated in cancer. Nature Communications. 2023;14(1):1975.

208. White EA. Manipulation of Epithelial Differentiation by HPV Oncoproteins. Viruses [Internet]. 2019; 11(4).

209. Choi J-H, Lee B-S, Jang JY, Lee YS, Kim HJ, Roh J, et al. Single-cell transcriptome profiling of the stepwise progression of head and neck cancer. Nature Communications. 2023;14(1):1055.

210. Romero-Masters JC, Lambert PF, Munger K. Molecular Mechanisms of MmuPV1 E6 and E7 and Implications for Human Disease. Viruses [Internet]. 2022; 14(10).

211. Doorbar J. Model systems of human papillomavirus-associated disease. The Journal of Pathology. 2016;238(2):166-79.

212. Cladel NM, Budgeon LR, Balogh KK, Cooper TK, Hu J, Christensen ND. Mouse papillomavirus MmuPV1 infects oral mucosa and preferentially targets the base of the tongue. Virology. 2016;488:73-80.

213. Wei T, Buehler D, Ward-Shaw E, Lambert PF. An Infection-Based Murine Model for Papillomavirus-Associated Head and Neck Cancer. mBio. 2020;11(3):10.1128/mbio.00908-20.

214. Bilger A, King RE, Schroeder JP, Piette JT, Hinshaw LA, Kurth AD, et al. A Mouse Model of Oropharyngeal Papillomavirus-Induced Neoplasia Using Novel Tools for Infection and Nasal Anesthesia. Viruses. 2020;12(4):450.

215. Tasoulas J, Srivastava S, Xu X, Tarasova V, Maniakas A, Karreth FA, et al. Genetically engineered mouse models of head and neck cancers. Oncogene. 2023.

216. Tasoulas J, Srivastava S, Xu X, Tarasova V, Maniakas A, Karreth FA, et al. Genetically engineered mouse models of head and neck cancers. Oncogene. 2023;42(35):2593-609.

217. Carper MB, Troutman S, Wagner BL, Byrd KM, Selitsky SR, Parag-Sharma K, et al. An Immunocompetent Mouse Model of HPV16(+) Head and Neck Squamous Cell Carcinoma. Cell Reports. 2019;29(6):1660-74.e7.

218. Henderson S, Chakravarthy A, Su X, Boshoff C, Fenton Tim R. APOBEC-Mediated Cytosine Deamination Links PIK3CA Helical Domain Mutations to Human Papillomavirus-Driven Tumor Development. Cell Reports. 2014;7(6):1833-41.

219. Nichols AC, Palma DA, Chow W, Tan S, Rajakumar C, Rizzo G, et al. High Frequency of Activating PIK3CA Mutations in Human Papillomavirus–Positive Oropharyngeal Cancer. JAMA Otolaryngology–Head & Neck Surgery. 2013;139(6):617-22.

220. Chaturvedi AK, Graubard BI, Broutian T, Pickard RKL, Tong Z-Y, Xiao W, et al. Effect of Prophylactic Human Papillomavirus (HPV) Vaccination on Oral HPV Infections Among Young Adults in the United States. Journal of Clinical Oncology. 2017;36(3):262-7.
